# Endoscopic ultrasound–guided intratumoral alpha radiotherapy: clinical implementation of a novel therapeutic delivery platform

**DOI:** 10.1055/a-2971-5104

**Published:** 2026-10-12

**Authors:** Corey S Miller, Magali Lecavalier-Barsoum, David Roberge, Anand V. Sahai, Kim Ma, Field F. Willingham, Harold Jacob

**Affiliations:** 1 Division of Gastroenterology 5621 Jewish General HospitalMontrealCanada; 2 Division of Radiation Oncology 5621 Jewish General HospitalMontrealCanada; 3 Division of Radiation Oncology 25443 Centre Hospitalier de l'Université de MontréalMontrealCanada; 4 Division of Gastroenterology 25443 Centre Hospitalier de l'Universite de MontrealMontrealCanada; 5 Division of Medical Oncology 5621 Jewish General HospitalMontrealCanada; 6 Division of Digestive Health and Liver DiseasesDepartment of Medicine 12235 University of Miami Miller School of MedicineMiamiFloridaUnited States; 7 Department of Gastroenterology 58884 Hadassah Medical CenterJerusalemJerusalemIsrael

Pancreatic ductal adenocarcinoma (PDAC) is the seventh leading cause of cancer death worldwide, with a 13% 5-year survival and most patients presenting with unresectable disease. 1​ ​2​ Endoscopic ultrasound (EUS) offers minimally invasive access to the pancreas and is the gold standard for PDAC diagnosis 3​ ; yet, EUS-directed therapeutic approaches have not been established in clinical practice. Intratumoral alpha radiotherapy delivers short-range, high-linear-energy-transfer alpha particles into tumors, achieving tumor-cell destruction while sparing adjacent structures, a property particularly relevant in the peripancreatic region. Diffusing alpha-emitters Radiation Therapy (Alpha DaRT; Alpha Tau Medical, Jerusalem, Israel) uses radium-224-impregnated metallic sources that release alpha-emitting daughter isotopes into the surrounding tumor. 4​ Preliminary results have been reported in PDAC. 5​

We describe the clinical implementation of EUS-guided intratumoral alpha radiotherapy ( Video 1 ). The delivery system comprises a glycerin-filled device that backloads a source into a 22-gauge fine-needle aspiration needle ( Fig. 1 ); an adjustable pusher that maintains the source at the needle tip for deployment ( Fig. 2 ); and a retraction handle preloaded with four 1-cm sources ( Fig. 3 ). The handle simultaneously advances the stylet and withdraws the needle, depositing each source at the original needle-tip position. An audible click confirms release. Deployment of up to four sources during one needle pass reduces the number of punctures required.

**Video 1** Step-by-step demonstration of EUS-guided intratumoral alpha radiotherapy, including source backloading, adjustable pusher and retraction handle deployment, and EUS-guided intratumoral insertion.


**Fig. 1:**
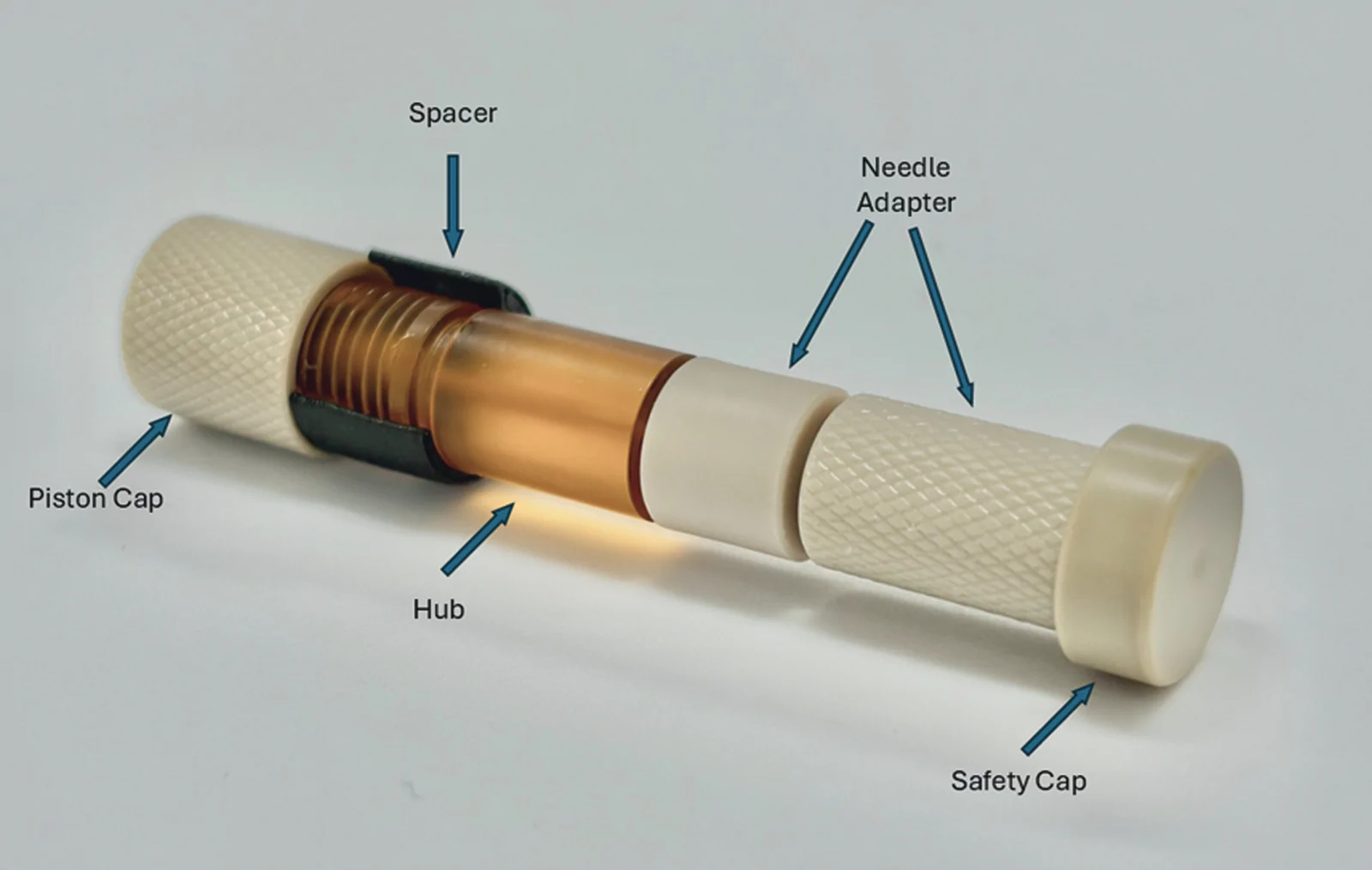
The Alpha DaRT loading device with labeled components: a piston cap, a hub (glycerin-filled), a spacer, a needle adaptor, and a safety cap. Each device is preloaded with a single radioactive source (1 or 2 cm in length) for direct use.

**Fig. 2:**
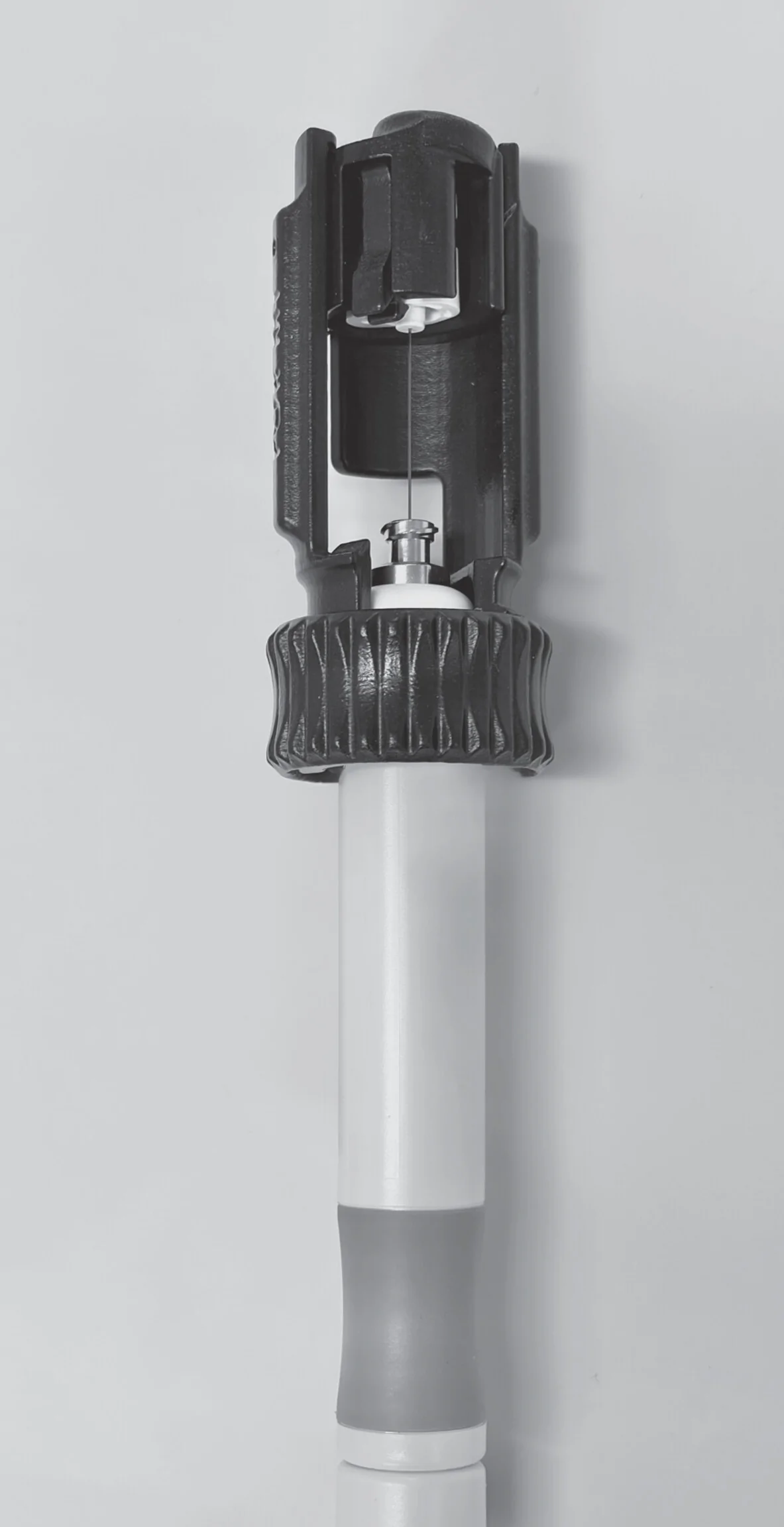
The adjustable pusher clipped onto a standard FNA needle handle. The pusher secures the stylet at a calibrated position corresponding to the source length loaded (1 or 2 cm), maintaining the source at the distal needle tip and enabling reliable deployment.

**Fig. 3:**
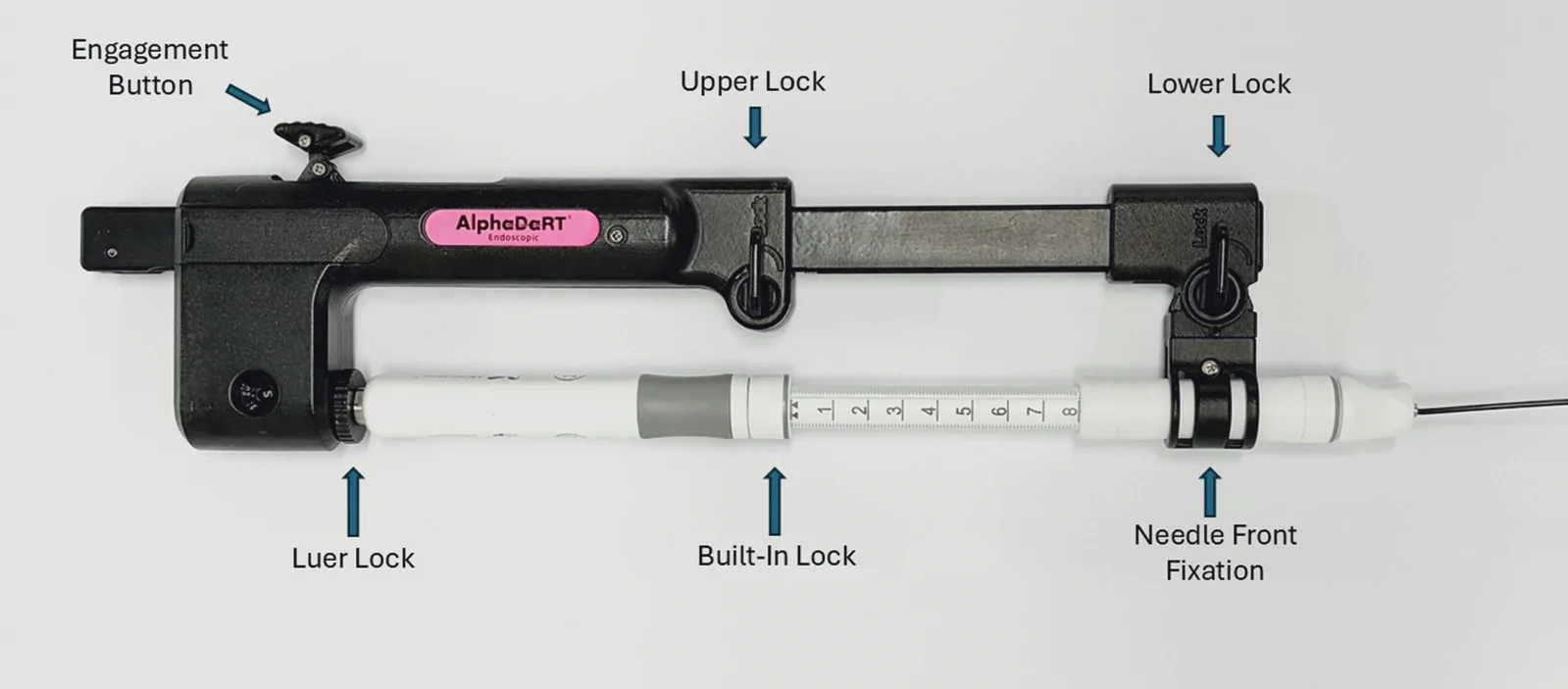
The purpose-designed retraction handle, preloaded with four 1-cm sources. Key components include the engagement button, upper lock (deployment mechanism), lower lock (needle movement), luer lock, built-in lock, and needle front fixation. Depression of the engagement button with the upper lock open simultaneously advances the stylet and retracts the needle by an equal and opposite distance, placing the source precisely at the original needle tip position.

Preprocedural computed tomographic (CT) volumetry determines the required number of sources, targeting 4-mm intersource spacing. Under real-time EUS guidance, color Doppler identifies intervening vessels before needle advancement. Sources are deployed sequentially from one or more scope positions until the planned distribution is achieved.

Representative EUS imaging during source insertion and CT imaging demonstrating complete radiological response are shown in Figs. 4 and 5 , respectively. This video provides a procedural reference for EUS-guided alpha radiotherapy, an emerging therapeutic platform with potential applicability across pancreatic cancer disease stages and other EUS-accessible tumor types. 5​

**Fig. 4:**
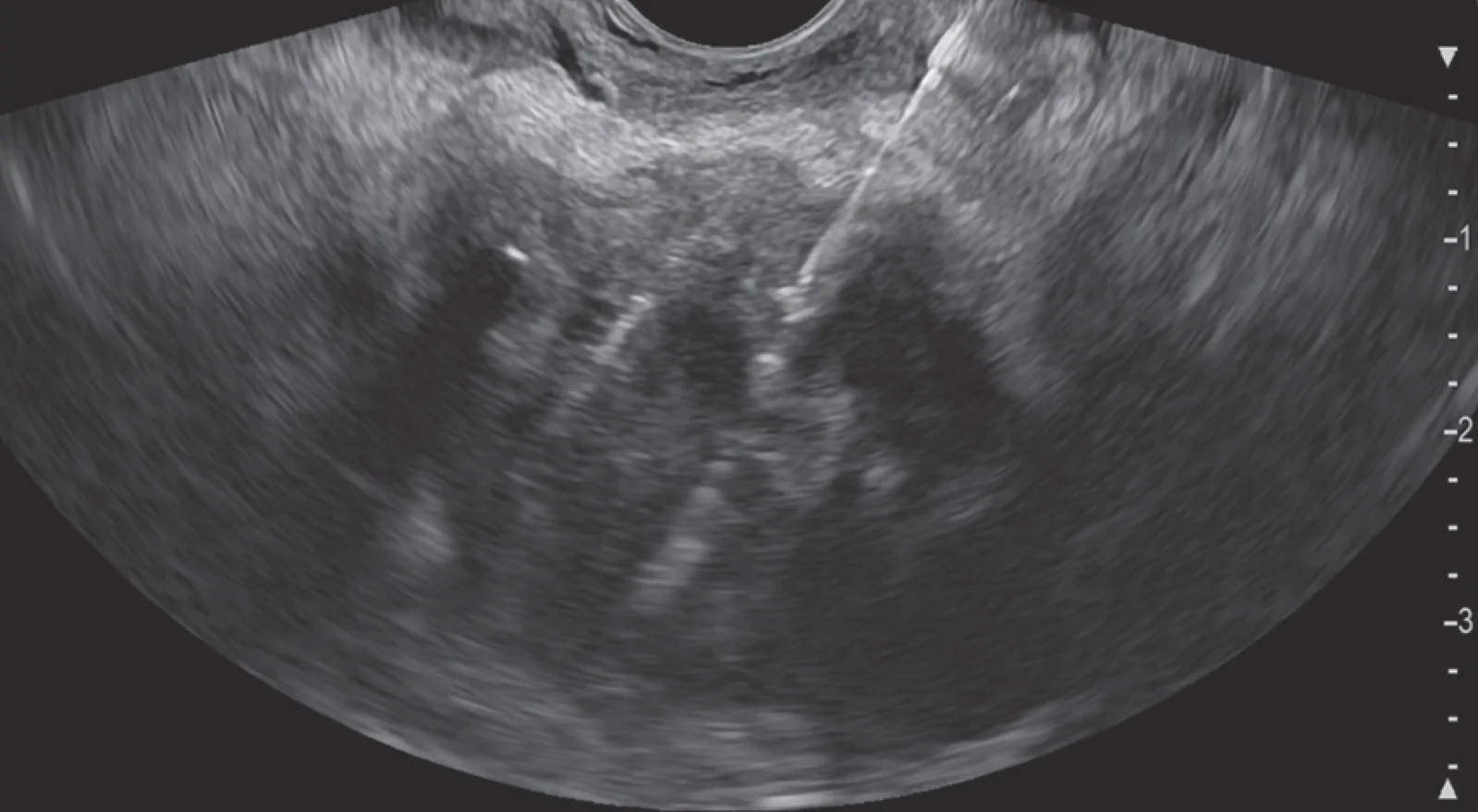
A representative EUS image during intratumoral alpha radiotherapy. A 22-gauge FNA needle is visualized within a hypoechoic pancreatic tumor, with hyperechoic artifact corresponding to an alpha DaRT source deployed in situ.

**Fig. 5:**
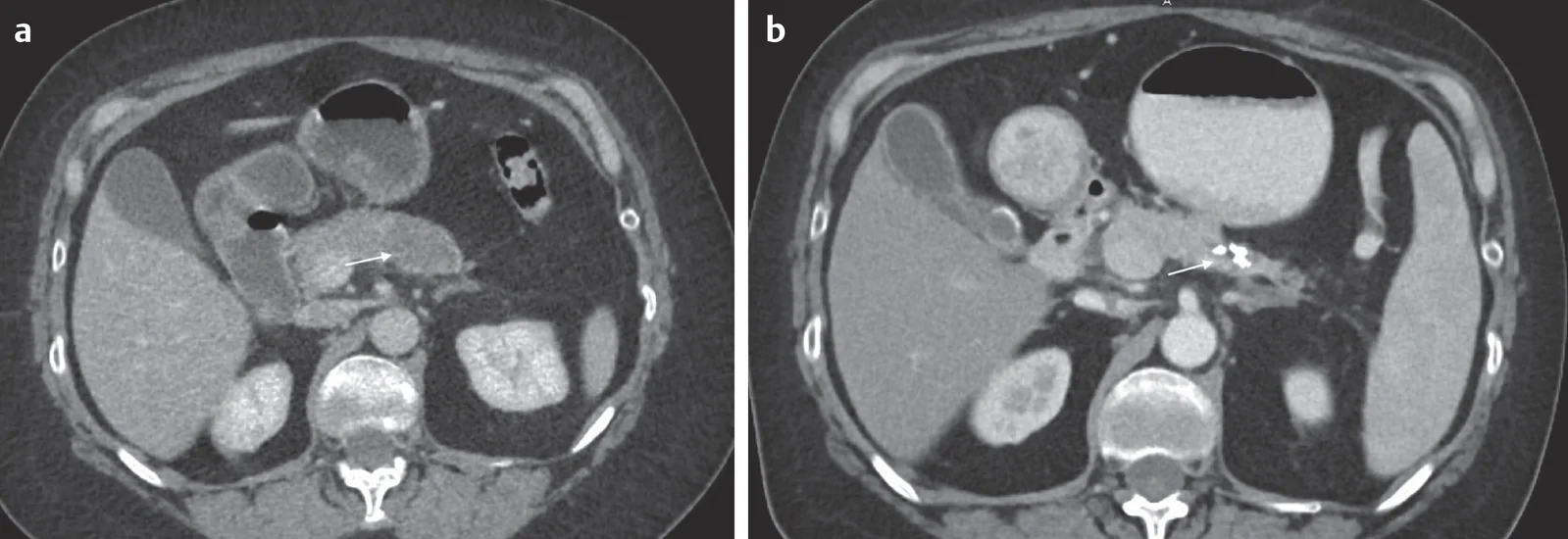
Representative CT imaging demonstrating complete radiological response following EUS-guided alpha radiotherapy. ( **a** ) Baseline axial CT showing the pancreatic tumor (white arrow). ( **b** ) Follow-up CT demonstrating the complete response of the primary tumor, with alpha DaRT sources visible in situ (white arrow).

Endoscopy_UCTN_Code_TTT_1AS_2AD

## Data Availability

No new data were generated or analyzed for this article.

## Declaration of GenAI Use

Claude (Anthropic) and ChatGPT (GPT-5.6 Sol; OpenAI) were used to assist with manuscript editing, formatting, and reference management. The authors reviewed and verified all contents and take full responsibility for the integrity of the work.
